# Catching the Culprit: How Chorea May Signal an Inborn Error of Metabolism

**DOI:** 10.5334/tohm.801

**Published:** 2023-10-06

**Authors:** Juan Darío Ortigoza-Escobar

**Affiliations:** 1 Department of Paediatric Neurology, Hospital Sant Joan de Déu, Barcelona, Spain; 2 European Reference Network for Rare Neurological Diseases (ERN-RND), Barcelona, Spain; 3 U-703 Centre for Biomedical Research on Rare Diseases (CIBER-ER), Instituto de Salud Carlos III, Barcelona, Spain

**Keywords:** Chorea, athetosis, ballismus, inborn errors of metabolism, neuroimaging

## Abstract

**Background:**

Movement disorders, particularly chorea, are uncommon in inborn errors of metabolism, but their identification is essential for improved clinical outcomes. In this context, comprehensive descriptions of movement disorders are limited and primarily derived from single cases or small patient series, highlighting the need for increased awareness and additional research in this field.

**Methods:**

A systematic review was conducted using the MEDLINE database and GeneReviews. The search included studies on inborn errors of metabolism associated with chorea, athetosis, or ballismus. The review adhered to PRISMA guidelines.

**Results:**

The systematic review analyzed 76 studies out of 2350 records, encompassing the period from 1964 to 2022. Chorea was observed in 90.1% of the 173 patients, followed by athetosis in 5.7%. Various inborn errors of metabolism showed an association with chorea, with trace elements and metals being the most frequent. Cognitive and developmental abnormalities were common in the cohort. Frequent neurological features included seizures, dysarthria, and optic atrophy, whereas non-neurological features included, among others, facial dysmorphia and failure to thrive. Neuroimaging and biochemical testing played crucial roles in aiding diagnosis, revealing abnormal findings in 34.1% and 47.9% of patients, respectively. However, symptomatic treatment efficacy for movement disorders was limited.

**Discussion:**

This study emphasizes the complexities of chorea in inborn errors of metabolism. A systematic approach with red flags, biochemical testing, and neuroimaging is required for diagnosis. Collaboration between neurologists, geneticists, and metabolic specialists is crucial for improving early detection and individualized treatment. Utilizing genetic testing technologies and potential therapeutic avenues can aid in the improvement of patient outcomes.

## Introduction

In 2010, the Taskforce on Childhood Movement Disorders defined chorea as “*an ongoing random-appearing sequence of one or more discrete involuntary movements or movement fragments*” and athetosis as “*a slow, continuous, involuntary writhing movement that prevents maintenance of a stable posture*” [1]. Additionally, this taskforce defined ballism as “*chorea affecting proximal joints such as the shoulder or hip, resulting in large-amplitude movements, sometimes with a flinging or flailing quality*.” The timing, duration, and direction of these movements appear random, and each movement may have distinct beginning and ending points, but it can be difficult to distinguish between them because they frequently overlap or follow one another immediately. Consequently, the movements may appear to transition between different muscle groups and may involve various body parts, such as the trunk, neck, face, tongue, and extremities [2].

Lesions in a number of brain regions can cause these movement disorders. Chorea is frequently associated with conditions that affect the cerebral cortex, basal ganglia [3], cerebellum [4], and thalamus [5]. Athetosis is associated with lesions of the basal ganglia [6], whereas ballismus or hemiballismus is associated mainly with lesions of the subthalamic nucleus [7] but can also be associated with lesions of other basal ganglia structures [8].

In children, chorea can present as a symptom with various underlying causes [1]. A recent review (Ortigoza-Escobar, 2020) demonstrated a strong association between movement disorders, including chorea, and inborn errors of metabolism. These inborn errors of metabolism constitute a wide-ranging group of genetic disorders that impact metabolic pathways [9]. In the context of inborn errors of metabolism, movement disorders frequently manifest as a combination of distinct types. This complexity makes it difficult to predict the specific inborn errors of metabolism based solely on the manifestation of the movement disorder. Even when a specific type of movement disorder, such as chorea, is identified, multiple possibilities must still be considered, as some inborn errors of metabolism may involve a combination of different types of movement disorders. A recent review reveals that 51 percent of analyzed inborn errors of metabolism manifest with two or more movement disorders, whereas isolated movement disorders, such as mild ataxia or chorea, are uncommon and are usually part of a larger clinical picture with several non-neurological symptoms. 13% of the 231 inborn errors of metabolism considered in the study by Ortigoza-Escobar exhibited chorea [9].

In addition, the severity of movement disorders can evolve and worsen in the same patient over time. Despite accounting for less than one-fourth of movement disorder diagnoses in children, early identification of underlying inborn errors of metabolism is essential because it enables disease-specific treatments [10] and optimal outcomes for affected children and provides insights into the underlying pathophysiological mechanisms.

Comprehensive descriptions of movement disorders in inborn errors of metabolism are limited [11,12,13,14,15,16,17,18], with the majority of knowledge derived from single cases or small patient series. Even in patients initially believed to have acquired causes of movement disorders, such as cerebral palsy, it is crucial to consider the possibility of an inborn error of metabolism [19,20,21].

The primary objective of this review is to investigate the relationship between chorea and inborn metabolic error and to elucidate the clinical manifestations, diagnostic clues, and therapeutic implications of these metabolic diseases in the context of chorea by examining the existing literature and analyzing relevant clinical cases.

## Methods

A systematic review following the Preferred Reporting Items for Systematic Reviews and Meta-Analyses (PRISMA) checklist was conducted to ensure methodological rigor [22]. The detailed description of the methodology for the literature search can be found in the Supplemental Data. Methods.

“*Dyskinesia*” is the medical term for a variety of involuntary, abnormal, and frequently repetitive movements or muscle contractions, sometimes used synonymously with “hyperkinetic movement disorders”, whereas in other instances, it refers to chorea or to both chorea and dystonia [23]. These movements can manifest in a variety of ways, such as twitching, jerking, writhing, or slow and sustained motions. It is important to note that some of the articles included in this review use the term “dyskinesia” to refer to chorea [24].

## Results

### Studies selection

The search strategy identified a total of 2350 records. Following a full-text review of 143 articles, 76 studies were included in this systematic review based on the selection criteria. A visual representation of the study selection process is provided in Figure S1, while Tables S1 and S2 list all included studies after full-text review.

### Studies characteristics

The 76 studies included in this review [16,24,25,26,27,28,29,30,31,32,33,34,35,36,37,38,39,40,41,42,43,44,45,46,47,48,49,50,51,52,53,54,55,56,57,58,59,60,61,62,63,64,65,66,67,68,69,70,71,72,73,74,75,76,77,78,79,80,81,82,83,84,85,86,87,88,89,90,91,92,93,94,95] were published between 1964 [39] and 2022 [80] and were conducted in various countries (Table S2). Of these, 30 were case reports, and 46 were case series.

In the case series studies included in this review, the number of patients varied significantly. The study with the highest number of patients included was conducted by Kalita et al., 2021, which included 31 patients [84]. The detailed characteristics of these studies are presented in Table 1.

**Table 1:** Included case series studies reporting prevalence of chorea in cohort of Inborn Errors of Metabolism.


REFERENCE	DISORDERS	CATEGORY OF DISORDERS BASED ON THE ICIMD CLASSIFICATION.	SUBCATEGORY OF DISORDERS BASED ON THE ICIMD CLASSIFICATION.	TYPE OF ARTICLE	PATIENTS WITH CHOREA (PERCENTAGE OF TOTAL PATIENTS REPORTED IN THE ARTICLES WHO EXHIBIT THIS SYMPTOM)

François Haude et la., 2022 [1]	3-hydroxyisobutyryl-CoA hydrolase deficiency (HIBCH) and Mitochondrial short-chain enoyl-CoA hydratase 1 deficiency (ECHS1)	1. DISORDERS OF AMINO ACID METABOLISM	1.2 Organic acidurias	Case series	HIBCH 1 out of 24 patients (4%) and ECHS110 out of 61 patients (16%)

Ktena et al., 2015 [2]	Methylmalonic Acidemia	1. DISORDERS OF AMINO ACID METABOLISM	1.2 Organic acidurias	Case series	13 patients

Dreifuss et al., 2008 [3]	Hypoxanthine guanine phosphoribosyltransferase deficiency (Lesch-Nyhan syndrome)	16. DISORDERS OF NUCLEOBASE, NUCLEOTIDE AND NUCLEIC ACID METABOLISM	16.2 Disorders of purine metabolism	Case series	29 out of 29 patients (100%)

Lam et al., 2017 [4]	N-glycanase 1 deficiency	18. CONGENITAL DISORDERS OF GLYCOSYLATION	18.5 Other disorders of glycan metabolism	Case series	12 out of 12 patients (100%)

Oates et al., 2008 [5]	GM2 gangliosidosis	20. DISORDERS OF COMPLEX MOLECULE DEGRADATION	20.1 Disorders of sphingolipid degradation	Case series	7 out of 36 patients (19%)

Kalita et al., 2021 [6]	Copper-transporting ATPase subunit beta deficiency (Wilson disease)	22. DISORDERS OF TRACE ELEMENTS AND METALS	22.1 Disorders of copper metabolism	Case series	31 out of 82 patients (38%)

Kalita et al., 2022 [7]	Copper-transporting ATPase subunit beta deficiency (Wilson disease)	22. DISORDERS OF TRACE ELEMENTS AND METALS	22.1 Disorders of copper metabolism	Case series	2 out of 20 patients (10%)

Machado et al., 2006 [8]	Copper-transporting ATPase subunit beta deficiency (Wilson disease)	22. DISORDERS OF TRACE ELEMENTS AND METALS	22.1 Disorders of copper metabolism	Case series	19 patients

Mihaylova et al., 2012 [9]	Copper-transporting ATPase subunit beta deficiency (Wilson disease)	22. DISORDERS OF TRACE ELEMENTS AND METALS	22.1 Disorders of copper metabolism	Case series	82 patients (10%)

Prashanth et al., 2004 [10]	Copper-transporting ATPase subunit beta deficiency (Wilson disease)	22. DISORDERS OF TRACE ELEMENTS AND METALS	22.1 Disorders of copper metabolism	Case series	1 out of 307 patients (0,3%)

Starosta-Rubinstein et al., 1987 [11]	Copper-transporting ATPase subunit beta deficiency (Wilson disease)	22. DISORDERS OF TRACE ELEMENTS AND METALS	22.1 Disorders of copper metabolism	Case series	3 out of 31 patients (9,6%)

Taly et al., 2007 [12]	Copper-transporting ATPase subunit beta deficiency (Wilson disease)	22. DISORDERS OF TRACE ELEMENTS AND METALS	22.1 Disorders of copper metabolism	Case series	24 of 282 patients (8,5%)

Youn et al., 2012 [13]	Copper-transporting ATPase subunit beta deficiency (Wilson disease)	22. DISORDERS OF TRACE ELEMENTS AND METALS	22.1 Disorders of copper metabolism	Case series	1 out of 45 patients (2%)

Ranjan et al., 2015 [14]	Copper-transporting ATPase subunit beta deficiency (Wilson disease)	22. DISORDERS OF TRACE ELEMENTS AND METALS	22.1 Disorders of copper metabolism	Case series	12 out of 34 patients (35%)


ICIMD: International Classification of Inherited Metabolic Disorders, available at http://www.iembase.org/.

Table 2 contains additional information from GeneReviews chapters on chorea in inherited metabolic disorders, providing insight into relevant metabolic disorders that were not identified by the initial PubMed search.

**Table 2:** GeneReviews Chapters on Chorea in Inborn Errors of Metabolism.


REFERENCE	DISORDERS	TYPE OF DISORDERS	TYPE OF DISORDERS	COMMENTARIES

Bindu et al., [106]	Isolated Sulfite Oxidase Deficiency	1. DISORDERS OF AMINO ACID METABOLISM	1.5 Disorders of the metabolism of sulfur-containing amino acids and hydrogen sulfide	Late-onset ISOD manifests between ages six and 18 months and is characterized by ectopia lentis (variably present), developmental delay/regression, movement disorder characterized by dystonia and choreoathetosis, ataxia, and (rarely) acute hemiplegia as a result of metabolic stroke. The clinical course may be progressive or episodic. In the episodic form encephalopathy, dystonia, choreoathetosis, and/or ataxia are intermittent.

Gregory et al., [107]	Pantothenate kinase 2 deficiency	21. DISORDERS OF VITAMIN AND COFACTOR METABOLISM	21.5 Disorders of pantothenate and CoA metabolism	PKAN is characterized by early-childhood onset of progressive dystonia, dysarthria, rigidity, and choreoathetosis. Pigmentary retinal degeneration is common. Atypical PKAN is characterized by later onset (age >10 years), prominent speech defects, psychiatric disturbances, and more gradual progression of disease.

Kurian et al., [108]	Dopamine transporter deficiency	23. NEUROTRANSMITTER DISORDERS	23.1 Monoamine neurotransmission	Classic DTDS. Infants typically manifest nonspecific findings (irritability, feeding difficulties, axial hypotonia, and/or delayed motor development) followed by a hyperkinetic movement disorder (with features of chorea, dystonia, ballismus, orolingual dyskinesia). Over time, affected individuals develop parkinsonism-dystonia characterized by bradykinesia (progressing to akinesia), dystonic posturing, distal tremor, rigidity, and reduced facial expression. Tetrabenazine and benzodiazepines may be useful in controlling chorea and dyskinesia in early stages of the disease.

Heimer et al., [109]	Mitochondrial enoyl-CoA reductase deficiency	14. DISORDERS OF LIPID METABOLISM	14.1 Disorders of fatty acyl synthesis, elongation, and recycling	*MECR*-related neurologic disorder is characterized by a progressive childhood-onset movement disorder and optic atrophy; intellect is often – but not always – preserved. The movement disorder typically presents between ages one and 6.5 years and is mainly dystonia that can be accompanied by chorea and/or ataxia. Over time some affected individuals require assistive devices for mobility.

Cohen et al., [110]	Mitochondrial DNA polymerase gamma catalytic subunit deficiency	9. DISORDERS OF MITOCHONDRIAL DNA MAINTENANCE AND REPLICATION	9.2 Disorders of mtDNA replication and maintenance	POLG-related disorders comprise a continuum of overlapping phenotypes. A POLG-related disorder should be suspected in individuals with combinations of the following clinical features and laboratory findings: Movement disorder (e.g., myoclonus, dysarthria, choreoathetosis, parkinsonism)


### Classification of Inborn Errors of Metabolism

**Figure 1:**
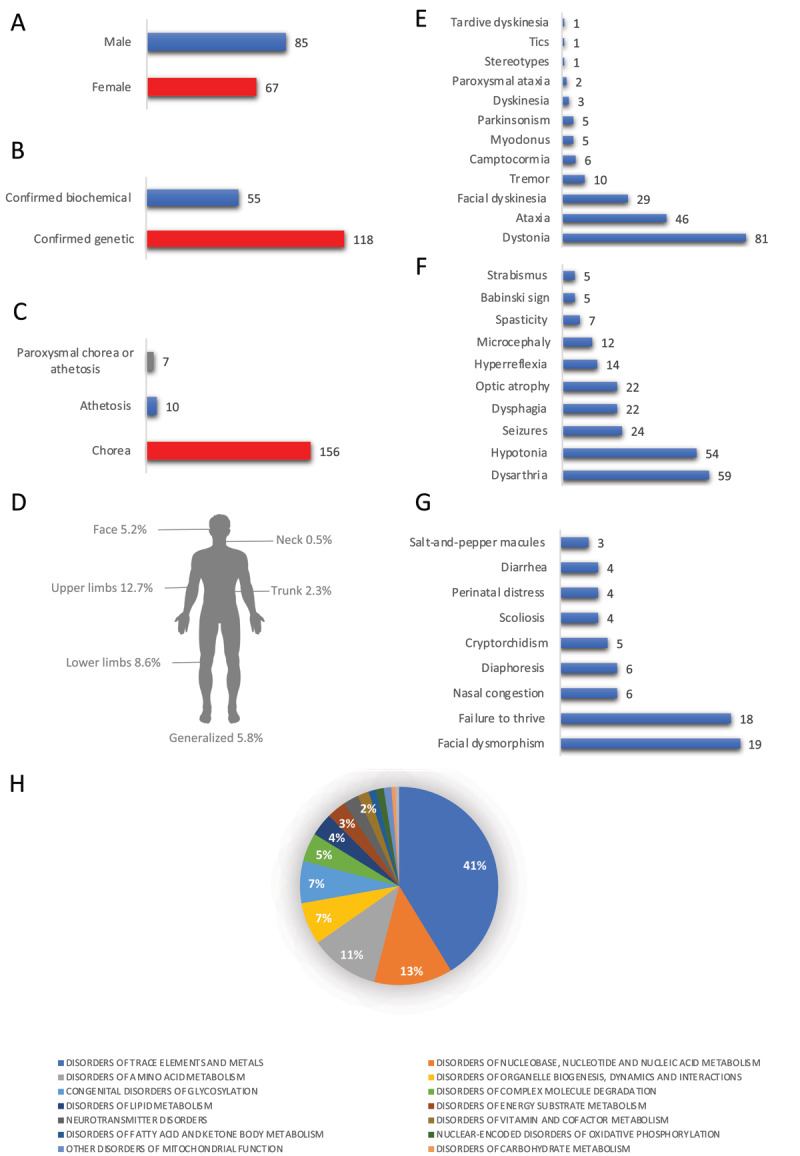
Overview of characteristics in patients with chorea and inborn errors of metabolism. **a)** Distribution by gender **b)** Distribution by genetically confirmed or biochemically diagnosed cases **c)** Distribution by types of movement disorders: chorea, athetosis, or paroxysmal disorders **d)** Frequency of chorea distribution e) Other associated movement disorders **f)** Other associated neurological symptoms **g)** Other associated non-neurological symptoms **h)** Distribution by specific types of inborn errors of metabolism.

Inborn errors of metabolism associated with chorea are diverse, with varying frequencies across different metabolic categories. Among the reported disorders, those related to trace elements and metals showed the highest frequency, with 132 cases documented. Disorders of nucleobase, nucleotide, and nucleic acid metabolism (41 cases), as well as disorders of amino acid metabolism (36 cases) and disorders of organelle biogenesis, dynamics, and interactions (36 cases), were also observed at relatively high frequencies (Figure 1H). The remaining metabolic categories exhibited lower frequencies, indicating a less common association with chorea.

### Patient Demographics and Clinical Characteristics

A total of 173 patients were included in the study, comprising 67 female (38.7%) patients, 85 male (49.1%) patients, and 21 (12.1%) cases where the gender was unspecified (Figure 1A). The mean age of the included patients was 21.45 ± 0.13 years (mean ± standard error), ranging from 2 months to 64 years. Among the cohort, 90 patients (52%) were under the age of 18. A total of 20 patients (11.5%) did not have their age specified.

Among the cohort of patients, 118 individuals (68.2%) were identified to have a confirmed genetic inborn error of metabolism, indicating the presence of underlying genetic abnormalities. Additionally, 55 patients (31.7%) displayed biochemical findings consistent with specific disorders, suggesting the presence of a specific inborn error of metabolism (Figure 1B). It is noteworthy that these patients were diagnosed during the era prior to the advent of genetic testing, highlighting the reliance on biochemical analyses for diagnosis in that time period.

### Chorea, athetosis and ballismus

All patients included in this review exhibited specific continuous or intermittent movement disorders. Chorea was present in 156 patients (90.1% of the cohort). Athetosis was observed in 10 patients (5.7%), including glutaric aciduria type 1, Lesch Nyhan syndrome and AADC deficiency (Aromatic L-amino acid decarboxylase deficiency) [35,37,57,67]. Seven patients (4.0%) displayed intermittent episodes of both chorea and athetosis [40,58,65,69,76,79], including those with nonketotic hyperglycinemia, neuronal ceroid lipofuscinosis, hereditary hemochromatosis type 1, GABA transaminase deficiency and ornithine transcarbamylase deficiency (Figure 1C).

The age of onset of chorea was not available for 125 patients (72.2% of the cohort). Among the remaining patients, the mean age of chorea onset was 9.32 ± 0.32 years (mean ± standard error, range 3 months to 63 years). In 39 of these patients (22.5%), chorea manifested before the age of 18, and in ten (5.7%) patients, the onset occurred before the age of one year [25,26,27,39,46,48,60,71,96]. These patients had the following inborn errors of metabolism: 3-methylglutaconic aciduria, galactose-1-phosphate uridylyltransferase deficiency, beta-ketothiolase deficiency, Lesch-Nyhan syndrome, Leigh syndrome, OPA3 deficiency, 6-pyruvoyl-tetrahydropterin synthase deficiency, multiple carboxylase deficiency and succinic semialdehyde dehydrogenase deficiency.

The data on the distribution of chorea in the included patients revealed a variety of patterns; consequently, the distribution of chorea cannot be ascribed to a specific inborn error of metabolism. The most common localizations were limbs and generalized chorea, reported in multiple cases. Facial involvement was also observed (e.g., glutaric aciduria type 1, mitochondrial ATP synthase F0 subunit 6 deficiency, *OPA3* deficiency, metachromatic leukodystrophy, 6-pyruvoyl-tetrahydropterin synthase deficiency and hereditary ceruloplasmin deficiency), often in combination with other regions such as the limbs, trunk, or neck. Tongue involvement was noted in some patients, including those with glutaric aciduria type 1, mucopolysaccharidosis type 2, metachromatic leukodystrophy and succinic semialdehyde dehydrogenase deficiency. Additionally, there were reports of paroxysmal episodes in GABA transaminase deficiency involving the neck, arms, and trunk, accompanied by drowsiness and triggered by fever or hot weather [69]. Chorea distribution information was unavailable for 129 (74.5%) individuals. The distribution of chorea in the published cases is shown in Figure 1D.

The most prevalent associated movement disorder was dystonia, observed in 81 patients (46.8% of the total). Paroxysmal ataxia in 2 patients (1.1%), and ataxia in 46 patients (26.5%). Tremor was observed in 10 patients (5.7%), myoclonus in 5 patients (2.8%), and stereotypes in 1 patient (0.5%). Tics were reported in 1 patient (0,5%), while parkinsonism was observed in 5 patients (2.8%). Camptocormia was found in 6 patients (3.4%). Facial dyskinesia, including mainly orolingual dyskinesia was observed in 29 patients (15.5%) while one patient (0,5%) exhibited generalized “dyskinesia” (Figure 1E).

Oculogyric crisis was observed in seven patients (4.0%), including patients with 6-pyruvoyl-tetrahydropterin synthase deficiency and aromatic L-amino acid decarboxylase deficiency.

### Other neurological and non-neurological features

Other than movement disorders, 154 (90.0%) individuals had available data on other neurological features. The most prevalent neurological symptoms were hypotonia (54; 31.2%), seizures (24; 13,8%), and dysarthria (59; 34.1%). Other commonly reported neurological features included optic atrophy (22; 12.7%), dysphagia (22; 12.7%), and microcephaly (12; 6.9%). Other neurological symptoms are shown in Figure 1F.

Out of the total, 9 individuals (5.3%) exhibited normal cognition, while the majority presented with various cognitive and developmental abnormalities. Specifically, cognitive regression, developmental delay (including both motor and language delay), and motor regression were observed in the remaining cases. Among these cases, 11 individuals (6.3%) demonstrated severe cognitive impairment.

125 (72.2%) individuals lacked data on additional non-neurological symptoms. In 19 (10.9%) patients, facial dysmorphism was the most prevalent symptom, followed by failure to thrive in 18 (10.4%) patients. Other notable symptoms included scoliosis (Guanidinoacetate methyltransferase deficiency, *ALG6*-CDG, *PMM2*-CDG and neuronal ceroid lipofuscinosis), brachydactyly (*ALG8*-CDG), joint laxity (*ALG8*-CDG and Mucopolysaccharidosis type 2), diarrhea (*ALG8*-CDG, *PMM2*-CDG and aromatic L-amino acid decarboxylase deficiency), cryptorchidism (*ALG8*-CDG, *ALG6*-CDG, *PMM2*-CDG and *COG5*-CDG), and kidney stones (Lesch-Nyhan syndrome), each observed in multiple patients. Additional symptoms such as hepatomegaly (galactose-1-phosphate uridylyltransferase deficiency, *COG5*-CDG and *PMM2*-CDG), hypertrophic cardiomyopathy (dihydrolipoamide dehydrogenase deficiency), pulmonary embolism (Lesch-Nyhan syndrome), urinary tract infections (Lesch-Nyhan syndrome), and optic atrophy (*OPA3* deficiency, neuronal ceroid lipofuscinosis, phosphatidylserine flippase deficiency and 3-methylglutaconic aciduria) were also present in the cohort (Figure 1G).

### Neuroimage

Normal neuroimaging was observed in 12 (6.9%) individuals [29,35,47,77,78,94], including individuals with galactose-1-phosphate uridylyltransferase deficiency, *SUCLG1* deficiency, Lesch-Nyhan syndrome, *OPA3* deficiency, phosphatidylserine flippase deficiency, and Birk-Landau-Perez syndrome. Neuroimages were unavailable for 102 (58.9%) individuals. In the remaining 59 (34.1%) individuals, abnormal neuroimaging findings (CT or brain MRI) were observed (Table 3).

**Table 3:** Inborn Errors of Metabolism Associated with Chorea Distributed by Neuroimaging Abnormalities.


NORMAL NEUROIMAGE	GENERAL BRAIN OBSERVATIONS	CEREBELLUM	WHITE MATTER	BASAL GANGLIA	OTHERS

Galactose-1-phosphate uridylyltransferase deficiency [94] SUCLG1 deficiency [29] Hypoxanthine guanine phosphoribosyltransferase deficiency (Lesch-Nyhan syndrome) [35] OPA3 deficiency [47] Birk-Landau-Perez syndrome [78]	*Enlarged basal cisterns and Sylvian fissures* Glutaryl-CoA dehydrogenase deficiency [24] *Brain atrophy* GLUT1 deficiency [95] a-ketoglutarate dehydrogenase deficiency [27] Dihydrolipoamide dehydrogenase deficiency [28] Niemann-Pick C disease [53] Iduronate sulfatase deficiency (Mucopolysaccharidosis type 2) [54] Neuronal Ceroid Lipofuscinosis [58] Biotinidase deficiency [27] GABA transaminase deficiency [70] FBXL4 deficiency [74] Mitochondrial NAD kinase 2 deficiency [75] Phosphatidylserine flippase deficiency [77]	*Enhancement of cerebellar folia* Nonketotic hyperglycinemia [90] *Cerebellar atrophy* ALG6-CDG [90] ALG8-CDG [90] PIGN-CDG [19] PMM2-CDG [90] Hereditary ceruloplasmin deficiency [64] *Dentate nuclei hyperintensity* COG5-CDG [90] *Dentate nuclei hypointensity* Hereditary hemochromatosis type 1 [65]	*Subcortical white matter hyperintensity* Mitochondrial tRNA-Leu 1 deficiency [31] ALG8-CDG [90] PMM2-CDG [90] COG5-CDG [90] Multiple carboxylase deficiency [27] Succinic Semialdehyde Dehydrogenase Deficiency [71] FBXL4 deficiency [74] Mitochondrial NAD kinase 2 deficiency [75] Birk-Landau-Perez syndrome [78] *Hypomyelination* GABA transaminase deficiency [70] *Delayed myelination* Creatine transporter deficiency [72] Phosphatidylserine flippase deficiency [77]	*Putamen and caudate hyperintensity* Beta-Ketothiolase Deficiency [26] Mitochondrial ATP synthase F0 subunit 6 deficiency [27] NADH dehydrogenase alpha subcomplex subunit 10 deficiency [32] VPS13D deficiency [51] Multiple carboxylase deficiency [27] Copper-transporting ATPase subunit beta deficiency (Wilson disease) [62,61] FBXL4 deficiency [74] Mitochondrial NAD kinase 2 deficiency [75] Ornithine transcarbamylase deficiency [76] *Putamen and caudate hypointensity* Hereditary ceruloplasmin deficiency [64] *Globus palidus hyperintensity* Copper-transporting ATPase subunit beta deficiency (Wilson disease) [62,61] Succinic Semialdehyde Dehydrogenase Deficiency [71] Creatine transporter deficiency [72] *Thalamic hyperintensity* Nonketotic hyperglycinemia [79] a-ketoglutarate dehydrogenase deficiency [27] COG5-CDG [90] Copper-transporting ATPase subunit beta deficiency (Wilson disease) [62,61] GABA transaminase deficiency [69] *Thalamic hypointensity* Hereditary ceruloplasmin deficiency [64]	*Peduncles hyperintensity* Beta-Ketothiolase Deficiency [26] Copper-transporting ATPase subunit beta deficiency (Wilson disease) [62,61] *Dilated basal ganglia Virchow–Robin spaces* ALG8-CDG [90] *Pons atrophy* PMM2-CDG [90] *Hypoplastic optic nerve* Phosphatidylserine flippase deficiency [77] *Thin corpus callosum* Creatine transporter deficiency [72] Phosphatidylserine flippase deficiency [77] *MRS increase Cr peak* Guanidinoacetate methyltransferase deficiency [30] *MRS absence Cr peak* Creatine transporter deficiency [72] *MRS increase lactate peak* Mitochondrial tRNA-Leu 1 deficiency [31] *MRS increase GABA peak* GABA transaminase deficiency [69] Succinic Semialdehyde Dehydrogenase Deficiency [71]


In certain cases, neuroradiological assessment should encompass other areas such as the optic nerves and meninges, as abnormalities were observed in these regions. MR spectroscopy was useful to confirm the diagnosis in cases of creatine deficiency syndrome [72], guanidinoacetate methyltransferase deficiency [30], mitochondrial tRNA-Leu 1 deficiency [31], succinic semialdehyde dehydrogenase deficiency [71], and GABA transaminase deficiency [69].

### Biochemical testing

In 88 (50.8%) patients, there were no results indicating abnormalities in the biochemical tests, and 2 (1.1%) patients showed normal results in the biochemical tests. In the remaining 83 (47.9%) patients, some abnormalities in the biochemical tests were observed (Table S1). These laboratory results collectively contribute to the diagnostic evaluation and characterization of the cohort with inborn errors of metabolism.

**Figure 2:**
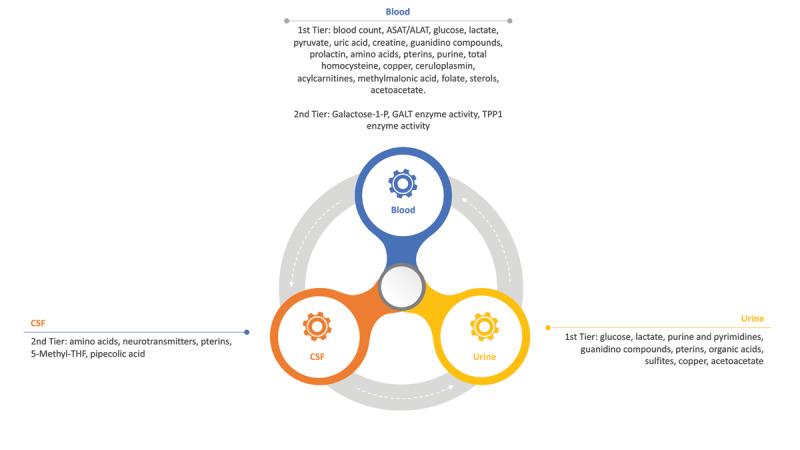
displays the essential minimum laboratory tests recommended for patients presenting with chorea and suspected movement disorders. ASAT: Aspartate Aminotransferase, ALAT: Alanine Aminotransferase, TPP1: Tripeptidyl Peptidase 1, GALT: Galactose-1-Phosphate Uridyltransferase, 5-Methyl-THF: 5-Methyltetrahydrofolate and Galactose-1-P: Galactose-1-Phosphate.

Figure 2 displays the essential minimum laboratory tests recommended for patients presenting with chorea and suspected inborn errors of metabolism.

### Treatment

The treatment of chorea in the reviewed patients varied considerably. Some individuals refused treatment [27], while others received specific interventions. Pharmacological interventions to reduce chorea included carbamazepine, haloperidol, midazolam, corticosteroids, gamma-vinyl-GAB, clonazepam, trazodone, L-dopa/benserazide, baclofen, trihexyphenidyl, medical cannabis, botulinum toxin, venlafaxine, clozapine, tetrabenazine, MAO inhibitors, and dopamine agonists. Some patients showed improvement with these treatments [27], while others experienced a partial effect [30] or no response. Treatments for chorea are shown in Table S1. Given the lack of specific recommendations regarding when to initiate chorea treatment, which medication to use, and at what dosage, as well as the lack of extensive studies describing chorea treatments in inborn errors of metabolism, the choice of treatment was likely based on clinical experience.

Among the cohort, 52 (30.0%) patients were documented to have undergone treatment pertaining to the inborn error of metabolism. The treatment status of 119 (68.7%) patients remains unspecified, failing to provide information on the specific interventions received. It was observed that two (1%) patients did not receive any form of treatment.

The treatment approach for the cohort in this study was diverse and tailored to the specific conditions and needs of each individual. The disease-specific treatments encompassed dietary modifications such as a low-lysine and low-tryptophan diet, a ketogenic diet with L-carnitine, protein restriction, and specific supplementation with vitamins and cofactors like biotin, thiamine, L-carnitine, and riboflavin. Additionally, other treatments included enzyme replacement therapy and chelating agents. Table 4 presents disease-specific treatments for inborn errors of metabolism presenting with chorea.

**Table 4:** Disease-specific treatment for inborn errors of metabolism presenting with chorea.


DISORDERS	TREATMENT

Mitochondrial disorders	Mitochondrial cocktail (may include biotin, thiamine, coenzyme Q10, riboflavin, carnitine, etc)

Glutaryl-CoA dehydrogenase deficiency	Adherence to emergency protocol in infancy and early childhood. Carnitine 100 mg/kg/d. Diet (Lys and Trp-restricted)

Nonketotic hyperglycinemia	Experimental dextromethorphan (5–20 mg/kg/day), Na-benzoate (250 – 750 mg/kg/day), folinic acid (15 mg/day)

Galactose-1-phosphate uridylyltransferase deficiency	Lactose-free infant formula, lactose-free, galactose-restricted diet

GLUT1 deficiency	Ketogenic diet. Avoidance of certain drugs (barbiturates, ethanol, methylxanthines, and tricyclic antidepressants)

Guanidinoacetate methyltransferase deficiency	Creatine 400 mg/kg/day, ornithine supplementation 100–800 mg/kg/day + arginine restriction 15–25 mg/kg/day

Niemann-Pick C disease	Substrate inhibition therapy (Miglustat), HSCT in early diagnosed patients with NPC2 mutations

Metachromatic leukodystrophy	HSCT in pre-symptomatic or early symptomatic

Multiple carboxylase deficiency	Biotin 10–40 mg/day

Partial biotinidase deficiency	Biotin 5–10mg/day

Wilson disease	Avoid copper in food and drinking water, zinc, trientene, D-penicillamine, liver transplantation

Hereditary hemochromatosis type 1	Ifiron overload/symptoms: regular phlebotomy

6-pyruvoyl-tetrahydropterin synthase deficiency	BH4(1–20 mg/kg/day), L-dopa/carbidopa (2–11 mg/kg/day) and 5-hydroxytryptophan (0.5–8.5 mg/kg/day)

Aromatic L-amino acid decarboxylase deficiency	Bromocriptine, trihexyphenidyl, pergolide, tranylcypromine, vitamin B6, MAO inhibitors, gene therapy

Succinic Semialdehyde Dehydrogenase Deficiency	Symptomatic, including methylphenidate, thioridazine, risperidone and BZD

Ornithine transcarbamylase deficiency	Acute inpatient treatment: maintain anabolic state, limit protein intake, arginine 100 – 200 mg/kg/day, ammonia remotion: Na-benzoate 250 – 400 mg/kg/day or Na-phenylbutyrate 250 – 500 mg/kg/day,

Neuronal Ceroid Lipofuscinosis	CLN2: Cerliponase alfa.


### Methodological quality of the included studies

The results of the evaluation of methodological quality revealed that, out of the total number of articles analyzed, 7 (11.4%) were rated good, 38 (62.2%) were rated moderate, and 16 (26.2%) were rated poor (Table S3). This evaluation allowed for an assessment of the validity and rigor of the included studies. There was relatively less evidence and room for improvement in the detailed description of the distribution of chorea and the documentation of associated non-neurological symptoms. These findings highlight potential gaps in the existing literature, indicating a need for more thorough reporting on these particular topics.

## Discussion

This is the first comprehensive review to analyze systematically the genetic, clinical, and treatment responses of 173 patients with inborn errors of metabolism presenting with chorea, athetosis, or ballismus. The data were extracted from an extensive screening of over 76 scientific articles. Although the number of included patients is not high, it is essential to note that the conditions under study are rare diseases. Thus, the number of participants is considered optimal, as they come from diverse backgrounds and exhibit varying clinical characteristics.

### The evaluation of movement disorders suspected of having a metabolic origin should include consideration of neurological and non-neurological features, biochemical studies, and radiological findings as essential components for identifying treatable causes

When evaluating movement disorders suspected of having a metabolic cause, it is essential to consider a wide range of hyperkinetic and hypokinetic manifestations. Due to the potential overlap of clinical characteristics among various diseases, diagnosing these disorders can be difficult. Therefore, a comprehensive evaluation system is essential to accurately identify and diagnose these disorders. The evaluation of a child should start with red flags, including neurological and non-neurological features, found in Ortigoza-Escobar, 2020 [9] that serve as crucial indicators for further investigation, including the characterization of the movement disorder phenotype, neurological symptoms, and biochemical studies to identify treatable diseases. In addition, radiological findings and genetic testing are essential for confirming the diagnosis. Similar diagnostic algorithm schemes have been developed for adult-onset chorea [97]. The ultimate objective of this evaluation is to enable the prompt detection of treatable inborn errors of metabolism, ensuring timely and disease-specific treatments to improve patient outcomes.

### Chorea, athetosis, or ballismus are rare movement disorders caused by inborn errors of metabolism, typically presenting in combination with other movement disorders

The co-occurrence of dystonia and chorea, known as choreoathetosis, is a well-documented phenomenon in various inborn errors of metabolism. Additionally, “dyskinesia”, paroxysmal ataxia, and ataxia were reported in a subset of patients, reflecting the heterogeneity of movement disorders in this context. As “*dyskinesia*” represents a medically imprecise term, it is strongly recommended that future descriptions employ more standardized terminology such as chorea, athetosis, or ballism.

The presence of paroxysmal ataxia suggests transient episodes of ataxia that may be associated with metabolic disturbances or other underlying mechanisms [79]. Tremor, myoclonus, and tics were less frequently reported, emphasizing their relative rarity in comparison to dystonia and ataxia. The presence of parkinsonism [61,63], and camptocormia [78] further highlights the diverse clinical manifestations of inborn errors of metabolism involving movement disorders. Hemiballismus, or ballism, is a rare manifestation of an inborn error of metabolism. It has been described in numerous cases of non-ketotic hyperglycemia, Wilson disease [87] and infrequently in some patients with Pantothenate kinase 2 deficiency [98]. It is essential to note that non-ketotic hyperglycemia is not considered an inborn error of metabolism itself; rather, it is a metabolic disorder characterized by very high blood sugar levels without the presence of ketones in the blood or urine. However, its clinical features may overlap with those of an inborn error of metabolism, posing diagnostic difficulties; thus, it is listed as a differential diagnosis.

### Conditions resembling chorea, athetosis, and ballismus should be approached with caution

Myoclonus is characterized by sudden, brief, shock-like involuntary movements caused by muscular contractions or inhibitions, while chorea is a nonpatterned, continuous movement disorder that appears flowing or jerky. The jerky components of chorea can be challenging to differentiate from myoclonus. In some cases, electroencephalography (EEG) and electromyography (EMG) recordings may be required for a precise diagnosis of the underlying movement disorder. *GOSR2*-CDG is a notable example of a disorder of vesicular trafficking that falls under the larger category of organelle biogenesis, dynamics, and interactions [99]. In cases where the clinical presentation closely resembles chorea or other hyperkinetic movements, it is essential for clinicians to consider the possibility of overlap between different movement disorders and use electrophysiological methods to ensure an accurate diagnosis.

Additionally, it is essential to note that nutritional deficiencies, such as serum vitamin B12 deficiency and hyperhomocystinemia [100], can mimic inborn errors of metabolism. Furthermore, non-ketotic hyperglycemia can also present with symptoms of chorea or hemiballismus [101], as mentioned previously.

### Chorea, athetosis, or ballismus typically manifest along with other neurological and non-neurological symptoms in individuals with developmental delay or cognitive decline

A small percentage of the reviewed cases exhibited normal cognition, while the majority exhibited a variety of cognitive and developmental abnormalities. Commonly observed were cognitive regression, developmental delay (including motor and language delays), and motor regression. In certain cases, children with chorea exhibited hypotonia, which contributed to motor delay. In addition, motor regression was occasionally accompanied by metabolic decompensation and basal ganglia injury [36]. In both adults (e.g., Niemann-Pick type C, aceruloplasminemia) [53,63] and children (e.g., ceroid lipofuscinosis and dihydrolipoamide dehydrogenase deficiency) [28,58], chorea was either preceded or accompanied by cognitive regression in certain cases.

### Chorea, athetosis, or ballismus are rarely the initial symptoms of an inborn error of metabolism

In the majority of cases, patients present with preceding features such as hypotonia and motor delay, and the onset of movement disorders, including chorea, typically occurs later in the disease course. Chorea, though observed in some cases at an early age, usually does not dominate the clinical picture initially. For instance, conditions like 3-methylglutaconic aciduria [25], galactose-1-phosphate uridylyltransferase deficiency [94], beta-ketothiolase deficiency [26], Lesch-Nyhan syndrome [39], Leigh syndrome [46], *OPA3* deficiency [48], 6-pyruvoyl-tetrahydropterin synthase deficiency [60], multiple carboxylase deficiency [27], and succinic semialdehyde dehydrogenase deficiency [71] may exhibit chorea at an early stage. In some specific cases, the movement disorder can indeed be the initial neurological sign, as observed in Niemann-Pick type C [52].

### The sudden onset of chorea, athetosis, or ballismus in the context of an encephalopathy should raise the suspicion of an inborn error of metabolism

During febrile illnesses, nonketotic hyperglycinemia has been associated with episodes characterized by lethargy, ataxia, marked chorea involving the head, trunk, and extremities, and confusion (delirium) [79]. Similarly, GABA transaminase deficiency has been associated with paroxysmal episodes of chorea in the neck, arms, and trunk, often accompanied by drowsiness, and these episodes are triggered by fever or hot weather [69]. Another illustrative example is ornithine transcarbamylase deficiency, which can manifest with encephalopathy and episodic ataxia [76]. These clinical case scenarios emphasize the need for a high index of suspicion for inborn errors of metabolism in patients presenting with sudden-onset movement disorders, especially when encephalopathy and other neurological manifestations are present. Therefore, a systematic approach to biochemical testing and neuroimaging, including MR spectroscopy when appropriate, is recommended when evaluating patients with acute or episodic movement disorders. Chorea can manifest in a paroxysmal manner, as described in cases of late-onset nonketotic hyperglycinemia with *GLDC* variants [90].

### Neuroimaging aids in the diagnosis of patients with chorea, athetosis, or ballismus and an inborn error of metabolism. A normal neuroimaging study does not, however, rule out the possibility of an inborn error of metabolism

Among patients for whom neuroimaging was available, various patterns of abnormalities were observed. Neuroimaging can be nonspecific, particularly in cases of cerebral atrophy, despite the fact that these findings may raise suspicion for certain metabolic disorders that are inherited. In some cases, neuroimaging provided information, particularly in the identification of cerebellar atrophy, white matter lesions, or hyperintensities in the basal ganglia, which can be indicative of specific metabolic disorders. In addition, abnormalities in the optic nerves were observed, highlighting the need for a comprehensive imaging evaluation that takes into account multiple brain structures. In certain conditions, like methylmalonic acidemia, movement disorders can be observed even in patients with apparently normal neuroimaging [81]. The utility of MR spectroscopy in confirming the diagnosis of specific metabolic disorders such as creatine deficiency syndrome, guanidinoacetate methyltransferase deficiency, mitochondrial tRNA-Leu 1 deficiency, succinic semialdehyde dehydrogenase deficiency, and GABA transaminase deficiency.

In addition, the presence of basal ganglia hyperintensities accompanied by chorea in the context of encephalopathy should raise the suspicion of organic acidurias, specifically glutaric aciduria due to its relative frequency, or a mitochondrial disorder. In contrast, the presence of lesions in the basal ganglia, involvement of the thalamus, and signs of hepatopathy in children should prompt consideration of Wilson disease. Patients with multisystemic manifestations, including both neurological and non-neurological features, as well as cerebellar atrophy, should raise suspicions of a glycosylation defect.

### When assessing chorea, athetosis, or ballismus caused by a suspected inborn error of metabolism, it is helpful to use a systematic approach to biochemical testing

This systematic approach allows for a comprehensive assessment of metabolic abnormalities, which can be critical in identifying underlying conditions even when some routine analyses appear normal. Certain patients may harbor inherited metabolic disorders (e.g., *OPA3* deficiency, [47]) despite initially unremarkable biochemical testing. Moreover, metabolic abnormalities may only manifest during periods of metabolic decompensation, as in Leigh syndrome [46], emphasizing the importance of repeated testing and monitoring over time. In addition, certain inborn errors of metabolism require specialized analyses, such as cerebrospinal fluid (CSF) examinations, as for nonketotic hyperglycinemia [79] and GABA transaminase deficiency [70] individuals, or enzymatic activity studies in fibroblasts, as for arylsulfatase A deficiency [55], GM2 gangliosidosis [56], and neuronal ceroid lipofuscinosis [58] individuals, for accurate diagnosis and characterization.

### Disorders of trace elements and metals appear to result in chorea more often than other types of disorders

Several diseases, such as Wilson disease, hereditary ceruloplasmin deficiency, ferritin light chain superactivity, and Birk-Landau-Perez syndrome, are characterized by disturbances in trace element and metal metabolism. This disruption frequently results in the accumulation of these elements, particularly in the basal ganglia. The basal ganglia exhibits heightened sensitivity to changes in trace elements and metals, making it susceptible to functional disruptions when imbalances occur. Furthermore, it is important to note that historical factors may have contributed to the higher reported incidence of chorea in conditions such as Wilson disease. For instance, Wilson disease has been identified and studied for a considerable amount of time, which may have resulted in a larger number of documented cases of chorea. The availability of biomarkers for this disorder is widespread. Additionally, the prevalence of Wilson disease, one of the most prevalent inherited metabolic disorders, contributes to the higher incidence of chorea associated with this condition. The consistent presence of choreiform movements in patients across diverse clinical settings and multiple research studies strengthens the previously established connection between trace element and metal-related disorders and the development of chorea.

### Symptomatic treatment for chorea, athetosis, or ballismus associated with inborn errors of metabolism is rarely highly effective

Out of the cohort, only 30% of reported patients had documented treatment specifically targeting the underlying inborn error of metabolism. However, for the majority of patients, the treatment status remains unspecified, leaving crucial details such as dosages, administration methods, and treatment outcomes unreported. Furthermore, the effectiveness of the medications used is not assessed using standardized scales, and the adverse effects of the medications are not discussed. Additionally, alternative therapeutic options, such as deep brain stimulation or gene therapy, are not mentioned, and the potential impact of treating the underlying inborn error of metabolism on the movement disorders is not explored. The lack of comprehensive information on symptomatic treatment in the available literature highlights the need for more in-depth studies and standardized assessments to better understand and improve the management of these complex cases.

### Utilizing genetic testing to optimize patient outcomes

When specific biomarkers are absent or when neuroimaging findings can be attributed to multiple metabolic disorders, genetic tests are useful for the diagnosis of inborn errors of metabolism [102,103]. However, it is essential to recognize that the timeliness of genetic testing can limit its usefulness, particularly in acute cases. Nonetheless, as precision medicine and gene therapy continue to develop, genetic testing is anticipated to grow in importance in the future. Not only does it aid in diagnosis, but it also has the potential to guide individualized therapeutic interventions, making it an integral part of meaningful genetic counseling.

### Early initiation of disease-specific treatment of inborn errors of metabolism can prevent the worsening of chorea

In the context of inborn errors of metabolism, early initiation of disease-specific treatment is crucial, especially when chorea is a prominent clinical manifestation. A timely intervention can significantly mitigate the progression and severity of chorea, as supported by evidence from clinical practice and scientific studies. In glutaric aciduria, early detection and treatment with a low-lysine diet and carnitine supplementation have been shown to prevent the neurologic complications, such as chorea, that can develop if the condition is left untreated [104]. Similarly, prompt administration of chelating agents such as D-penicillamine or zinc can effectively reduce copper accumulation and prevent or alleviate choreiform movements, hepatic dysfunction, and other Wilson disease clinical manifestations [105]. In fact, the use of a mitochondrial cocktail and general precautions, such as avoiding triggers (certain drugs, fever, strenuous physical exercise, trauma, etc.), can play a crucial role in preventing decompensations in mitochondrial diseases. These preventative measures are important because they can reduce the risk of basal ganglia injury and the progression of chorea.

### Therapeutic decision-making should consider the level of functional impairment due to chorea

In the field of movement disorders, therapeutic decision-making is frequently a difficult task. When disease-modifying therapies are unavailable, symptomatic treatments become the foundation of care. Nonetheless, it is essential to recognize that addressing the impact on functional impairment requires more than pharmacological interventions. The management of symptoms should be holistic, taking into account not only the relief of motor symptoms but also the prevention of secondary complications. In addition, the presence of comorbidities, such as psychiatric symptoms and intellectual disabilities, should influence therapeutic decisions. These decisions should be supported by a comprehensive risk-benefit analysis. The questions posed by Mohammad et al. are instructive in this regard: How effective is the treatment for this disease, and what is its risk-to-benefit ratio? Is the movement disorder the primary cause of impairment, or are other associated issues of greater concern? What is the risk of doing nothing? [10]. It is essential to note that there are no standardized guidelines for chorea management in specific contexts at present. Rather, these are consensus-based decisions that must be made jointly by healthcare providers and patients. In addition, it is essential to remain abreast of emerging treatments, such as gene therapy and other disease-modifying therapies, as they may provide future interventional opportunities.

### Strengths and limitations

This comprehensive review offers insights into the relationship between chorea, athetosis, and ballismus and inherited metabolic disorders, shedding light on their complexities and diagnostic considerations. The data, extracted from a comprehensive screening of numerous scientific articles, serves as a foundation for understanding these rare movement disorders. However, it is necessary to acknowledge several limitations that may impact the findings. These include the possibility of reporting bias affecting prevalence estimates, the predominance of case reports and small case series limiting generalizability, and the heterogeneity of study designs, patient characteristics, and treatment options, which makes it difficult to draw definitive conclusions. Moreover, the lack of standardized diagnostic criteria for some metabolic disorders and potential omissions in the literature search can affect the comprehensiveness of the results. Variations in methodological quality and the absence of long-term follow-up data in certain studies may introduce bias and influence treatment efficacy evaluation and disease progression assessment.

As a final limitation, the lack of specific searches for each particular inborn error of metabolism and the MeSH term [chorea] from the search criteria are acknowledged. This methodology may have resulted in the exclusion of relevant studies and data pertaining to rare metabolic disorders that cause chorea. Consequently, it is essential to recognize that the findings of the review may not exhaustively cover all relevant literature in the field. While this methodology allowed for a more comprehensive examination of chorea within the context of inherited metabolic disorders, it does introduce a bias toward more prominent or well-documented cases.

## Conclusions

This review provides insights into the complexities and diagnostic considerations surrounding inborn errors of metabolism manifesting as chorea, highlighting the need for further research and clinical awareness in this field. It is essential to recognize that some movement disorders, especially myoclonus, may resemble chorea. In addition, developmental delay and cognitive decline, as well as additional neurological and non-neurological features, are frequently observed in these patients. The sudden onset of chorea, athetosis, or ballismus in the context of encephalopathy should raise suspicion for an underlying inborn error of metabolism, whereas neuroimaging is a valuable diagnostic tool, although its ability to rule out these conditions is limited. Biochemical and genetic testing plays a pivotal role in diagnosis. The level of functional impairment should be considered when making therapeutic decisions, and early initiation of disease-specific treatment for inborn errors of metabolism can prevent the deterioration of the movement disorder.

## Data Accessibility Statement

The data that support the findings of this systematic review are available from the corresponding author upon reasonable request.

## Additional Files

The additional files for this article can be found as follows:

10.5334/tohm.801.s1 Supplemental Data. Methods.

10.5334/tohm.801.s2 Figure S1. PRISMA Flow Diagram of the Literature Search Process.

10.5334/tohm.801.s3 Table S1. provides a comprehensive compilation of clinical data for all patients included in this study from various case reports and case series with available information.

10.5334/tohm.801.s4 Table S2. Characteristics of Included Articles.

10.5334/tohm.801.s5 Table S3. Methodological quality of the included studies.
