# Supplementary material for: Catching the Culprit: How Chorea May Signal an Inborn Error of Metabolism
Source: Tremor Other Hyperkinet Mov (N Y). 2023 Oct 6;13:36. doi: 10.5334/tohm.801 (PMC10558026; doi:10.5334/tohm.801)
Supplement: Supplemental Data. — Methods. [file tohm-13-1-801-s1.pdf]

Supplementary Material. Methods.

Search strategy

A thorough search of the MEDLINE database was conducted. The search was performed on May 2, 2023, and updated thereafter on July 17, 2023, and included records of any date. The following search strategy was used: ("Inborn Error of metabolism”) AND ("chorea” OR “athetosis” OR “ballismus”). In addition, any additional relevant studies identified that may have been missed in the initial search were included based on a thorough examination of the full-text articles. Furthermore, to enhance the inclusivity of inborn errors of metabolism associated with chorea, findings were supplemented with relevant articles from GeneReviews, which were not originally retrieved in the final PubMed search. The selected articles were (1–6)

Of note, the literature search using these MeSH terms did not yield any articles on choreoacanthocytosis (*VPS13A*), which is now considered a disorder of organelle interplay. After careful consideration, it was decided not to include this disorder, despite its classification as an inborn error of metabolism in the International Classification of Inherited Metabolic Disorders (ICIMD). This decision was based on the fact that, generally, it does not align with the typical conception of an IEM.

Eligibility criteria

Inclusion criteria for this study included patients of any age with a diagnosis of chorea, athetosis, or ballismus and genetic or biochemical confirmation of an inborn error of metabolism. All inborn errors of metabolism described in the International Classification of Inherited Metabolic Disorders (ICIMD), available at http://www.iembase.org/, were included. Any study design except narrative reviews, conference articles, and editorials was included, and only Spanish and English articles were considered.

Study selection

Following the removal of duplicate records, a study selection process was conducted. To ensure consistency, a subset of studies was initially screened to establish a shared understanding of the inclusion and exclusion criteria. All studies were subsequently assessed based on their titles and abstracts, and those that did not meet the predetermined inclusion criteria were excluded. The full texts of the remaining studies were then reviewed.

Data extraction

A systematic and standardized approach was undertaken to extract pertinent data from the included studies. A pre-planned and well-designed data extraction sheet was utilized to extract all relevant characteristics of the studies. After conducting a preliminary evaluation of the first ten articles to identify the key characteristics that were consistently relevant and extractable across studies, this methodology was established. The extracted data included author, publication year, country of origin, targeted population characteristics, age at onset, distribution and treatment of chorea, athetosis or ballismus, accompanied movement disorder, neurological and non-neurological characteristics, neuroimage, biochemical testing, and treatment.

Methodological quality of the included studies

Multiple key characteristics were used to evaluate the methodological quality of the included studies. Each article was evaluated according to its study type, whether it described the age at onset of chorea and its distribution and treatment, and whether it reported on other movement disorders, additional neurological signs, non-neurological signs, neuroimaging, and laboratory testing of clinical cases. Each of these items was assigned one point by a scoring system. On the basis of their cumulative scores, the articles

were then divided into three categories: good (7-9 points), moderate (4-6 points), and poor (3 points). It is important to note that this evaluation was not performed on GeneReviews-sourced articles or descriptive cohort studies.

The scoring system used to evaluate the methodological quality of the included studies was employed primarily for internal assessment and categorization of the articles rather than as an exclusion criterion. Due to the scarcity of relevant literature in this field, it was essential to maximize data inclusion. While the scores were used to classify articles as being of good, moderate, or poor quality, they were not the sole criterion for study inclusion or exclusion. This evaluation instead served as an internal tool for classifying and differentiating the methodological quality of the included articles. It allowed for a more comprehensive understanding of the variety of studies and their quality levels within the scope of this systematic review.

References

1. Bindu P, Nagappa M, Bharath R, Taly A. Isolated Sulfite Oxidase Deficiency Summary Suggestive Findings. Gene Rev [Internet]. 2017;1–14.
2. Friedman J. Sepiapterin Reductase Deficiency. GeneReviews®. 1993;1–16.
3. Gregory A, Hayflick SJ. Pantothenate Kinase-Associated Neurodegeneration Summary GeneReview Scope Suggestive Findings. 2020;1–20.
4. Kurian MA. SLC6A3 -Related Dopamine Transporter Deficiency Syndrome Summary Genetic counseling GeneReview Scope. 2019;1–14.
5. Zeev B Ben. MECR -Related Neurologic Disorder Summary Clinical characteristics. 2023;

6. Cohen BH, Chinnery PF, Copeland WC. POLG -Related Disorders Summary Clinical characteristics Genetic counseling GeneReview Scope. GeneReviews. 2020;1–32.
