# Supplementary material for: Catching the Culprit: How Chorea May Signal an Inborn Error of Metabolism
Source: Tremor Other Hyperkinet Mov (N Y). 2023 Oct 6;13:36. doi: 10.5334/tohm.801 (PMC10558026; doi:10.5334/tohm.801)
Supplement: Figure S1. — PRISMA Flow Diagram of the Literature Search Process. [file tohm-13-1-801-s2.pdf]

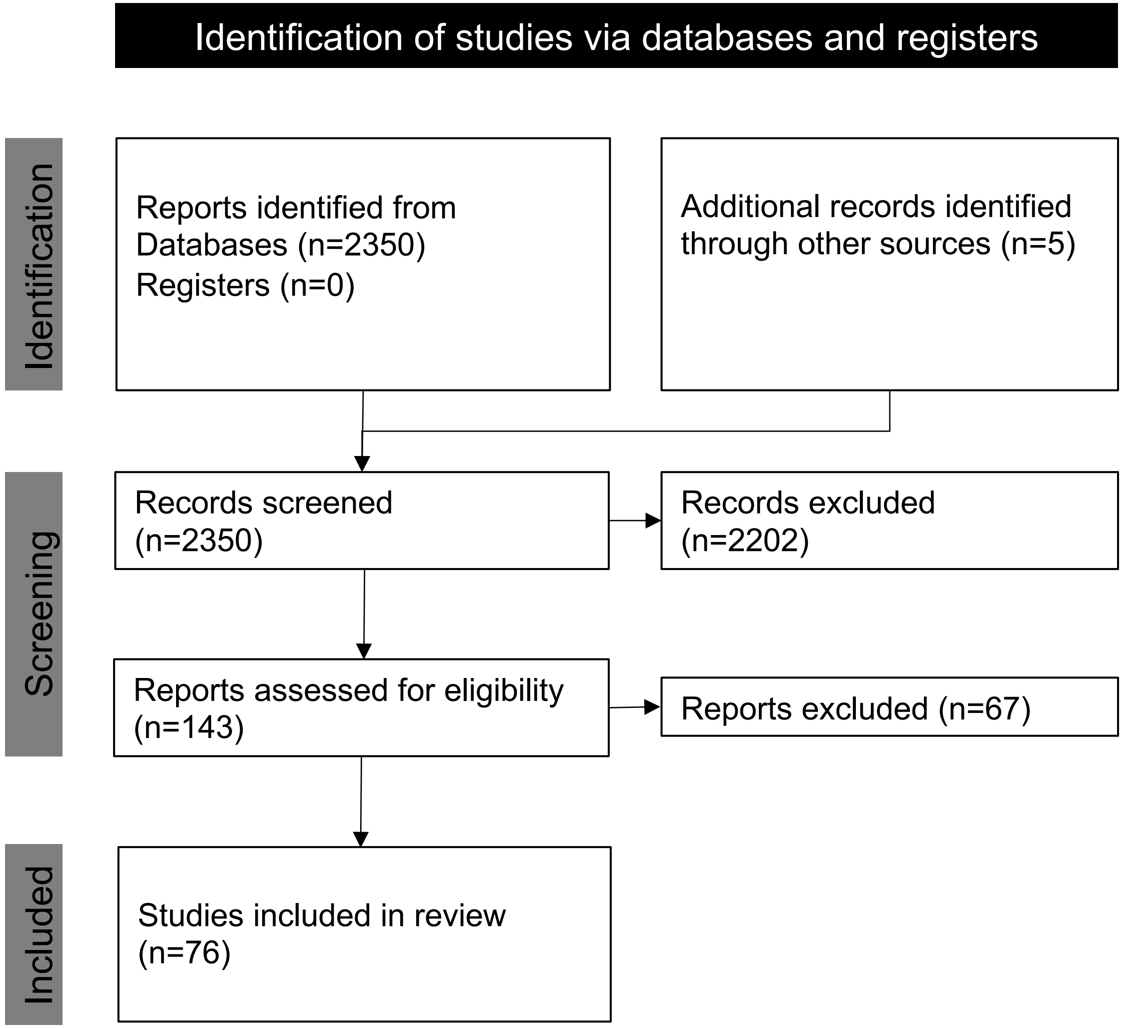

From: Page MJ, McKenzie JE, Bossuyt PM, Boutron I, Hoffmann TC, Mulrow CD et al. The PRISMA 2020 statement: an updated guideline for reporting systematic reviews. BMJ. 2021 Mar 29;372:n71.
