# Supplementary material for: Catching the Culprit: How Chorea May Signal an Inborn Error of Metabolism
Source: Tremor Other Hyperkinet Mov (N Y). 2023 Oct 6;13:36. doi: 10.5334/tohm.801 (PMC10558026; doi:10.5334/tohm.801)
Supplement: Table S1. — provides a comprehensive compilation of clinical data for all patients included in this study from various case reports and case series with available information. [file tohm-13-1-801-s3.pdf]

Table S1. Study characteristics				
Author, year	Country	Study Type	Patients included	Disorders
Greter et al., 1978	Sweden	Case series	2	3-Methylglutaconic Aciduria
Leibel et al., 1980	USA	Case report	1	Glutaryl-CoA dehydrogenase deficiency
Ojwang et al., 2001	South Africa	Case report	1	Glutaryl-CoA dehydrogenase deficiency
Brandt et al., 1978	Denmark	Case report	1	Glutaryl-CoA dehydrogenase deficiency
Goodman et al., 1975	USA	Case series	2	Glutaryl-CoA dehydrogenase deficiency
Gregersen et al., 1977	Denmark	Case series	2	Glutaryl-CoA dehydrogenase deficiency
Steiner et al., 1996	Canada	Case report	1	Nonketotic hyperglycinemia
Brenton et al., 2014	USA	Case report	1	Nonketotic hyperglycinemia due to glycine decarboxylase deficiency
Shah et al., 2009	India	Case report	1	Galactose-1-phosphate uridylyltransferase deficiency
Perez Dueñas et al., 2009	Spain	Case report	1	GLUT1 deficiency
Buha et al., 2013	Canada	Case report	1	Beta-Ketothiolase Deficiency
Gascon et al., 1994	Saudi Arabia	Case series	1	a-ketoglutarate dehydrogenase deficiency.
Odièvre et al., 2005	France	Case series	3	Dihydrolipoamide dehydrogenase deficiency
Donti, 2011	USA	Case report	1	GTP-specific succinyl-CoA synthetase subunit alpha deficiency (SUCLG1 deficiency)
Sempere et al., 2009	Spain	Case series	1	Guanidinoacetate methyltransferase deficiency
Gascon et al., 1994	Saudi Arabia	Case series	1	Mitochondrial ATP synthase F0 subunit 6 deficiency
Lahiri et al., 2019	India	Case report	1	Mitochondrial tRNA-Leu 1 deficiency
Gascon et al., 1994	Saudi Arabia	Case series	3	Mitochondrial disease
Yahya et al., 2022	Italy	Case report	1	NADH dehydrogenase alpha subcomplex subunit 10 deficiency
Adler et al., 1996	USA	Case report	1	Hypoxanthine guanine phosphoribosyltransferase deficiency (Lesch-Nyhan syndrome)
Tsai et al., 2014	Taiwan	Case report	1	Hypoxanthine guanine phosphoribosyltransferase deficiency (Lesch-Nyhan syndrome)
Baba et al., 2016	Japan	Case report	1	Hypoxanthine guanine phosphoribosyltransferase deficiency (Lesch-Nyhan syndrome)
Berman et al., 1969	USA	Case series	2	Hypoxanthine guanine phosphoribosyltransferase deficiency (Lesch-Nyhan syndrome)
Kersnik Levart et al., 2007	Slovenia	Case report	1	Hypoxanthine guanine phosphoribosyltransferase deficiency (Lesch-Nyhan syndrome)
Lesch et al., 1964	USA	Case series	1	Hypoxanthine guanine phosphoribosyltransferase deficiency (Lesch-Nyhan

				syndrome)
Mitchell et al., 1984	Canada	Case report	1	Hypoxanthine guanine phosphoribosyltransferase deficiency (Lesch-Nyhan syndrome)
Mizuno et al., 1970	Japan	Case series	5	Hypoxanthine guanine phosphoribosyltransferase deficiency (Lesch-Nyhan syndrome)
Sorensen et al., 1970	USA	Case report	1	Hypoxanthine guanine phosphoribosyltransferase deficiency (Lesch-Nyhan syndrome)
Wood et al., 1972	USA	Case series	3	Hypoxanthine guanine phosphoribosyltransferase deficiency (Lesch-Nyhan syndrome)
Mostile et al., 2019	Italy	Case series	1	ALG6-CDG
Mostile et al., 2019	Italy	Case series	1	ALG8-CDG
Maydan et al., 2011	Israel	Case series	2	PIGN-CDG
Mostile et al., 2019	Italy	Case series	3	PMM2-CDG
Boccuto et al., 2014	USA	Case series	3	ST3GAL5-CDG
Mostile et al., 2019	Italy	Case series	1	Conserved oligomeric Golgi complex subunit 5 deficiency (COG5-CDG)
Macaya et al., 1993	USA	Case series	3	Leigh syndrome
Artif et al., 2013	Germany	Case series	2	OPA3 deficiency
Carmi et al., 2015	Israel	Case report	1	OPA3 deficiency
Neas 2005	Australia	Case series	1	OPA3 deficiency
Yahalom et al., 2014	Israel	Case series	18	OPA3 deficiency
Gauthier, 2018	Canada	Case series	4	VPS13D deficiency
Anheim et al., 2014	France	Case series	1	Niemann-Pick C disease
Shulman et al., 1995	USA	Case report	1	Niemann-Pick C disease
Farooq et al., 2008	USA	Case series	2	Iduronate sulfatase deficiency (Mucopolysaccharidosis type 2)
Kappler et al., 1991	Germany	Case series	3	Arylsulfatase A deficiency (Metachromatic leukodystrophy)
Oates et al., 1986	USA	Case report	1	GM2 gangliosidosis
Gahlot Saini et al., 2016	India	Case report	1	Late-Infantile Neuronal Ceroid Lipofuscinosis
Nass et al., 1986	USA	Case report	1	Neuronal Ceroid Lipofuscinosis
Roze et al., 2006	France	Case report	1	6-pyruvoyl-tetrahydropterin synthase deficiency
Gascon et al., 1994	Saudi Arabia	Case series	2	Multiple carboxylase deficiency
do Desterro Leiros da Costa, 2009	Brazil	Case series	5	Copper-transporting ATPase subunit beta deficiency (Wilson disease)
Hyeon Ahn, et al., 2020	Korea	Case series	1	Copper-transporting ATPase subunit beta deficiency (Wilson disease)
McNeill et al., 2008	Belgium	Case series	5	Hereditary ceruloplasmin deficiency

Yonekawa et al., 1999	Japan	Case report	1	Hereditary ceruloplasmin deficiency
Kumar et al., 2016	Canada	Case series	1	Hereditary hemochromatosis type 1
Russo et al., 2004	UK	Case report	1	Hereditary hemochromatosis type 1
Lee et al., 2009	Taiwan	Case series	6	Aromatic L-amino acid decarboxylase deficiency
Morales-Briceño et al., 2019	USA	Case series	2	GABA transaminase deficiency
Koening et al., 2017	The Netherlands	Case series	3	GABA transaminase deficiency
Zeiger et al., 2016	USA	Case report	1	Succinic Semialdehyde Dehydrogenase Deficiency
Anselm et al., 2006	USA	Case series	1	Creatine transporter deficiency
Chinnery et al., 2006	UK	Case series	28	Ferritin light chain superactivity
Gai et al., 2013	UK	Case series	3	FBXL4 deficiency
Houten et al., 2014	USA	Case report	1	Mitochondrial NAD kinase 2 deficiency
Keegan et al., 2003	USA	Case report	1	Ornithine transcarbamylase deficiency
McMillian et al., 2018	Canada	Case series	11	Phosphatidylserine flippase deficiency
Perez et al., 2017	Israel	Case series	6	Birk-Landau-Perez syndrome
François Haude et al., 2022	France	Case series	11	3-hydroxyisobutyryl-CoA hydrolase deficiency (HIBCH) and Mitochondrial short-chain enoyl-CoA hydratase 1 deficiency (ECHS1)
Ktena et al., 2015	USA	Case series	13	Methylmalonic Acidemia
Dreifuss et al., 2008	USA	Case series	29	Hypoxanthine guanine phosphoribosyltransferase deficiency (Lesch-Nyhan syndrome)
Lam et al., 2017	USA	Case series	12	N-glycanase 1 deficiency
Oates et al., 1986	USA	Case series	7	GM2
Kalita 2021	India	Case series	31	Copper-transporting ATPase subunit beta deficiency (Wilson disease)
Kalita 2022	India	Case series	2	Copper-transporting ATPase subunit beta deficiency (Wilson disease)
Machado et al., 2006	Brazil	Case series	19	Copper-transporting ATPase subunit beta deficiency (Wilson disease)
Mihaylova et al., 2012	Bulgaria	Case series	82	Copper-transporting ATPase subunit beta deficiency (Wilson disease)
Prashanth et al., 2004	India	Case series	1	Copper-transporting ATPase subunit beta deficiency (Wilson disease)
Starosta-Rubinstein et al., 1987	USA	Case series	2	Copper-transporting ATPase subunit beta deficiency (Wilson disease)
Taly et al., 2007	India	Case series	24	Copper-transporting ATPase subunit beta deficiency (Wilson disease)
Youn et al., 2012	Korea	Case series	1	Copper-transporting ATPase subunit beta deficiency (Wilson disease)
Ranjan et al., 2015	India	Case series	12	Copper-transporting ATPase subunit beta deficiency (Wilson disease)
